# Investigation into skin physiological parameters and mocroflora characteristics of melasma population in Lhasa China

**DOI:** 10.3389/fmicb.2025.1614050

**Published:** 2026-01-08

**Authors:** Longwei Fang, Bu Luo, Deqiong Danzen, La Yang, Yun Gao, Zhen Ni, Jin Wang, Zhuoma Duoji, Yang Ci, Wangdui Suolang, Wang Ge, Zhuoma Basang

**Affiliations:** 1 High Altitude Health Science Research Center, Tibet University, Lhasa, China; 2 Shigatse Tibetan Hospital, Shigatse, China; 3 Lhasa People’s Hospital, Lhasa, China; 4 Shigatse People’s Hospital, Shigatse, China

**Keywords:** Tibet, melasma, skin physiological characteristics, microbiome, flora characteristics

## Abstract

Melasma is a skin disease characterized by symmetrical pigmentation, mainly occurring in the exposed areas of the face, which has some relationship with skin parameters and bacterial diversity. High-altitude regions experience elevated ultraviolet (UV) radiation, potentially influencing skin parameters and microbial characteristics in melasma. To explore the inherent law of the melasma mechanism in high altitude, the hereditary Tibetans at high altitude in Lhasa, Tibet were investigated. Skin physiological parameters such as skin sebum (SM), corneum moisture content (CM), pH, trans epidermal water loss (TEWL), skin erythema index (E), melanin (M), individual typology angle (ITA) and so on, were measured and the microbiome characteristics were sequenced and analyzed. The results showed that among 302 participants, 36 were diagnosed with melasma (mean prevalence: 11.92%). Prevalence was significantly higher in females (16.67%) than males (2.04%), peaking in females aged 31–40 years (37.50%). Melasma patients exhibited significantly lower pH and higher M values compared to controls (*p* < 0.05). Community diversity analysis of alpha and beta of skin bacteria and fungi showed that the abundance, diversity, and flora composition in melasma population were basically the same as that of control population (*p* > 0.05). Species analysis of intergroup differences showed that the bacteria were dominated by the genera of Cutibacterium, Staphylococcus and so on, but it was no statistical significance, while Malassezia. Aspergillus, Aureobasidium, and Penicillium were the dominant genera of fungi at the genus level, with Aspergillus and Aureobasidium larger in the melasma group than the control group (*p* < 0.05). Correlation analysis showed that there were significant differences between the flora characteristics and the parameters of ITA, TEWL, pH, and E, M, SM, which may has the association with the formation of melasma. The ROC analysis assessing fungi such as Aspergillus, Aureobasidium as a predictor of melasma yielded an AUC of 0.798, 0.828, indicating fair discriminatory ability. These findings demonstrate that high altitude hereditary Tibetan melasma population in Tibet has diverse skin parameters and microflora structural features. The strong correlation between microflora characteristics, skin parameters, and melasma prevalence provides a new foundation for research on the prediction, prevention, and treatment of dermatological conditions in high-altitude populations.

## 1 Introduction

Melasma is a common hyperpigmentation disorder presenting as symmetrical, light-to-dark brown facial patches in a butterfly distribution (Ke et al., 2025; Liu et al., 2022). These patches are usually located on the forehead, cheeks, nose, chin, and upper lip (Almasi Ghale et al., 2025). While affecting aesthetics, melasma also causes some psychological damage to patient (Dange et al., 2025; Karadogan and Irer, 2025). Quality of life studies have shown that melasma has a more serious negative impact on emotional state, socialization, and daily life (Liu et al., 2023). Studies have shown that Asians, Indians, Latinos, Americans, and African Americans are more likely to develop melasma than other races. It is most common in Singapore, Brazil, India, Hispanic or Asian origin, which happen to be near the equator and have strong UV exposure (Belzer and Parker, 2023). Around 5–6 million women are affected with melasma in the United States and the prevalence in India is around 25% in high-risk patients creating a significant healthcare burden (Pandya et al., 2025). Onset typically occurs between 20 and 40 years, with a female-to-male ratio ranging from 4:1 to 39:1. Key risk factors include UV exposure, hormonal contraception, pregnancy, certain medications, and genetic predisposition (Ali and Al Niaimi, 2025). Although the increased incidence rate, there was not deeply investigated into the mechanism of melasma.

High altitude medicine refers to altitudes between 2,500 and 4,500 m as high altitude (Liu et al., 2021). Tibet is a high - altitude high altitude located in a region of high cold, low oxygen, strong winds, strong ultraviolet rays, and strong radiation (Santos et al., 2025; Singh et al., 2013). These factors, particularly heightened UV exposure, may increase melasma incidence and impair skin barrier function. With the development of microbiomics, it has been gradually realized that skin microbiome dysbiosis can lead to the development of skin diseases such as melasma (Liu et al., 2022). A significant increase in transiently resident bacteria (e.g., Micrococcus spp.) in melasma lesion areas has been demonstrated, indicating a possible association between the development of melasma and changes in the skin microbiota (Sun et al., 2022). Souza and Silva (2025) elucidated that the skin microbiome may influence the development and treatment of melasma by regulating inflammatory pathways and melanin metabolism. They conducted a literature review and further elucidated the existence of an association between skin ecological dysregulation and the pathogenesis of melasma. While for high altitude regions, the mean prevalence of melasma was 14.94% (Xu et al., 2025) and people living in the northwest, which has a dry climate and high altitude, showed lower level of total lipids, lower alpha diversity, and lower abundance of Malassezia (Kumari et al., 2023; Li et al., 2019; Schmeller et al., 2022; Tao et al., 2024; Zeng et al., 2017; Zhang et al., 2023). Despite the evidence linking high-altitude environmental stressors and skin microbiome changes to melasma, the exact interactions and mechanisms remain undefined, particularly in Tibetan populations exposed to extreme UV and climatic conditions, which has affected the in-depth research and treatment of melasma, hindering targeted interventions.

This study investigates differences in facial skin physiological parameters and microbial communities between melasma patients and controls in a high-altitude Tibetan population. We aim to elucidate relationships and develop predictive models to inform melasma prevention and treatment strategies in high altitude populations.

## 2 Materials and methods

### 2.1 Research object

Hereditary Tibetans volunteers (aged 18–80 years) in high altitude areas of Lhasa, Tibet (altitude of 3,650 m), who are aware of the study, sharing same altitude environment and same life-work condition, have signed an informed consent form and participate voluntarily. Excluded: (1) currently suffering from a disease and the disease is in the active phase; (2) patients with asthma or other chronic respiratory diseases under treatment; (3) allergic body/skin type; (4) people with inflammatory skin diseases or other skin diseases; (5) people who have applied any anti-inflammatory drugs to the test site in the last two months; (6) patients with immunodeficiency or autoimmune disease; (7) women who are breastfeeding, pregnant, or preparing for pregnancy; (8) those who are unable to complete the prescribed content as required by the test. Control subjects were age- and environment-matched for 18–80 years old, living in the same altitude environment and same life-work condition, for example that people had same working time inside or outside for almost same UV exposure, hadn’t been hormonal status, or skin care habits.

### 2.2 Research methods

#### 2.2.1 Physiological parameter testing

##### 2.2.1.1 Testing equipment

Sebumeter^®^ (SM), Corneometer^®^ (CM), Skin-pH-Meter^®^ (pH), Vapometer^®^ (TEWL), SkinColorCatch^®^ (E, M, ITA°, L, a, b*) (Delfin Technologies Ltd.). Height and Weight Measuring Instrument and other general physical examination standard anthropometric equipment.

##### 2.2.1.2 Skin physiological parameters testing

Procedure: After signing consent and recording demographics, subjects acclimatized for 30 min (21 ± 1 °C, 50 ± 5% RH). Facial (zygomatic) parameters were measured per manufacturer protocols. Subjects refrained from skincare/cosmetics ≥ 24 h and washing on test day. (Approved by Tibet University Ethics Committee). And melasma was diagnosed by standardized diagnostic method (Ke et al., 2025; Liu et al., 2022). The diagnosis could be made based on the patient’s medical history and typical clinical manifestations. Combined with non-invasive detection techniques such as slide pressure testing and Wood’s lamp examination, further staging and classification and MASI (Melasma Area and Severity Index) score could be conducted.

The main test parameters are shown in Table 1.

**TABLE 1:** Skin physiological parameters.

No	Parameter	Abbreviation	Instrument
1	Sebum secretio (μg/cm^2^)	SM	SebumScale
2	Skin pH	pH	pH meter
3	Corneum moisture content (a.u.)	CM	Corneometer SC
4	Transdermal water loss [g/(m^2^⋅h)]	TEWL	VAPOMETER
5	Erythema	E	SkinColarCatch
6	Melanin	M	
7	ITA° (°)	ITA	
8	L value	L	
9	a value	a	
10	b value	b	

#### 2.2.2 Skin microbiome testing

##### 2.2.2.1 Sample collection

Collection: Subjects refrained from morning face washing. A 3 × 3 cm area on the left cheek was swabbed with sterile saline-moistened cotton swabs by trained personnel. Swabs were stored at **−**80°C.

##### 2.2.2.2 DNA extraction and PCR amplification

DNA Extraction and Amplification: Genomic DNA extracted via CTAB method. 16S rRNA (V3–V4 region; primers 341F/806R) and ITS PCR amplification performed. Products purified, quantified, pooled.Library Prep and Sequencing: Libraries prepared (NEB Next^®^ Ultra DNA Library Prep Kit), quality-checked (Agilent 5400), and sequenced (Illumina platform, 250 bp paired-end).

Procedure: Total genome DNA from samples was extracted using CTAB method. DNA concentration and purity was monitored on 1% agarose gels. According to the concentration, DNA was diluted to 1 ng/μL using sterile water.

16S rRNA genes of distinct regions (16S V3–V4) were amplified used specific primer [341F (5′-CCTAYGGGRBGCASCAG-3′) 806R (5′-GGACTACNNGGGTATCTAAT-3′)]with the barcode. All PCR reactions were carried out with 15 μL of Phusion^®^ High-Fidelity PCR Master Mix (New England Biolabs); 2 μM of forward and reverse primers, and about 10 ng template DNA. Thermal cycling consisted of initial denaturation at 98 °C for 1 min, followed by 30 cycles of denaturation at 98 °C for 10 s, annealing at 50 °C for 30 s, and elongation at 72 °C for 30 s. Finally 72 °C for 5 min.

Mix same volume of 1XTAE buffer with PCR products and operate electrophoresis on 2% agarose gel for detection. PCR products was mixed in equidensity ratios. Then, mixture PCR products was purified with Universal DNA (TianGen, China).

##### 2.2.2.3 Libraries generated and illumina sequencing

Sequencing libraries were generated using NEB Next^®^ Ultra DNA Library Prep Kit (Illumina, United States) following manufacturer’s recommendations and index codes were added. The library quality was assessed on the Agilent 5400 (Agilent Technologies Co Ltd., United States). At last, the library was sequenced on an Illumina platform and 250 bp paired-end reads were generated.

##### 2.2.2.4 Bioinformatics analysis

The analysis was conducted by following the “Atacama soil microbiome tutorial” of Qiime2docs along with customized program scripts.^1^ Briefly, raw data FASTQ files were imported into the format which could be operated by QIIME2 system using qiime tools import program. Demultiplexed sequences from each sample were quality filtered and trimmed, de-noised, merged, and then the chimeric sequences were identified and removed using the QIIME2 dada2 plugin to obtain the feature table of amplicon sequence variant (ASV). The QIIME2 feature-classifier plugin was then used to align ASV sequences to a pre-trained GREENGENES 13_8 99% database (trimmed to the V3V4 region bound by the 338F/806R primer pair) to generate the taxonomy table. Any contaminating mitochondrial and chloroplast sequences were filtered using the QIIME2 feature-table plugin. Appropriate methods include ANCOM, ANOVA, Kruskal Wallis, LEfSe and DEseq2 were employed to identify the bacteria with different abundance among samples and groups. Diversity metrics were calculated using the core-diversity plugin within QIIME2. Feature level alpha diversity indices, such as observed OTUs, Chao1 richness estimator, Shannon diversity index, and Faith’s phylogenetics diversity index were calculated to estimate the microbial diversity within an individual sample. Beta diversity distance measurements, including Bray Curtis, unweighted UniFrac and weighted UniFrac were performed to investigate the structural variation of microbial communities across samples and then visualized via principal coordinate analysis (PCoA) and nonmetric multidimensional scaling (NMDS). PLS-DA (Partial least squares discriminant analysis) was also introduced as a supervised model to reveal the microbiota variation among groups, using the “plsda” function in R package “mixOmics.” Redundancy analysis (RDA) was performed to reveal the association of microbial communities in relation to environmental factors based on relative abundances of microbial species at different taxa levels using the R package “vegan.” Co-occurrence analysis was performed by calculating Spearman’s rank correlations between predominant taxa and the network plot was used to display the associations among taxa. In addition, the potential KEGG Ortholog (KO) functional profiles of microbial communities was predicted with PICRUSt. Unless specified above, parameters used in the analysis were set as default.

### 2.3 Methods of analysis

Skin parameter data and ROC curve analyzed (SPSS 17.0). Normally distributed data: independent *t*-test; non-normal: Mann-Whitney U test (significance: *p* < 0.05).

Skin microbiomics data were accomplished through the Micromeritics Alliance Bioscience Cloud Platform^2^ 10. The R software (R 4.2.1) package “vegan,” Redundancy Diagnostic Approach (RDA) was applied to reveal potential associations between microbial communities and skin parameters. *p* < 0.05 indicated statistical significance. The linear correlation analysis of the flora characteristics and skin parameters was performed by linear regression modeling using the Micromeritics Alliance Bioscience Cloud Platform and R software.

## 3 Results

### 3.1 Basic information and melasma prevalence

A total of 302 study subjects were enrolled. Among them, 266 people were healthy people, 96 were males and 170 were females, average age 37. A total of 36 people with chloasma were screened out, including two men and 34 women, and the average prevalence of melasma was 11.92%, with the prevalence in females (16.67%) being greater than the prevalence of melasma in males (2.04%). Female patients had the highest prevalence 37.50% at the age of 31–40 years (Table 2).

**TABLE 2:** Total number of people, people with melasma and prevalence among Tibetan population in Lhasa Tibet.

Age groups	Male population			Female population		
	Total number of people	Melasma people	Prevalence/%	Total number of people	Melasma person	Prevalence/%
≦ 30 years	3	0	0.00	26	3	11.54
31–40 years	12	1	8.33	40	15	37.50
41–50 years	36	0	0.00	58	12	20.69
51–60 years	32	1	3.13	54	4	7.41
61–70 years	11	0	0.00	18	0	0.00
≧ 71 years	4	0	0.00	8	0	0.00
Total	98	2	2.04	204	34	16.67

The skin characterization parameters of female patients aged 31–40, 41–50, and 51–60 years were selected. Compared with control group, and only in the age group of 31–40 years, the pH values of melasma patients were significantly lower than those of control group (5.93 vs. 6.22, *p* = 0.021), and the M values were significantly greater than those of control group (682.2 vs. 653.4, *p* = 0.023), as shown in Table 3. As they grew older, the differences of skin parameter gradually became more and more similar. Meanwhile, SM, CM, E of melasma people were greater, TEWL, ITA, L, a, b of melasma people were lower than control people, but there was no statistical significance (*p* > 0.05).

**TABLE 3:** Comparison of skin parameter characteristics between melasma patients and control population aged 31–40 years old.

Skin parameters	Parameter characteristics		*P*-value
	Melasma group	Control group	
SM (μg/cm^2^)	8.13 (8.43)	6.60 (7.85)	0.617
pH	5.93 (0.56)	6.22 (1.09)	**0.021**
CM	66.95 ± 21.28	51.94 ± 35.49	0.103
TEWL [g/(m^2^⋅h)]	20.48 (10.91)	37.00 (82.85)	0.595
E	470 (22.76)	452.91 (86.38)	0.823
M	682.2 ± 45.84	653.40 ± 31.17	**0.023**
ITA (°)	5.33 ± 17.64	12.36 ± 9.52	0.109
L	52.13 (5.42)	52.88 (8.27)	0.268
a	13.87 ± 3.81	14.24 ± 3.85	0.768
b	19.33 ± 4.85	19.44 ± 3.58	0.937

The bold values indicate that pH and M value between melasma patients and control population aged 31–40 years old has significant difference.

### 3.2 Skin microbial sequencing results

#### 3.2.1 Analysis of facial flora composition

**FIGURE 1:**
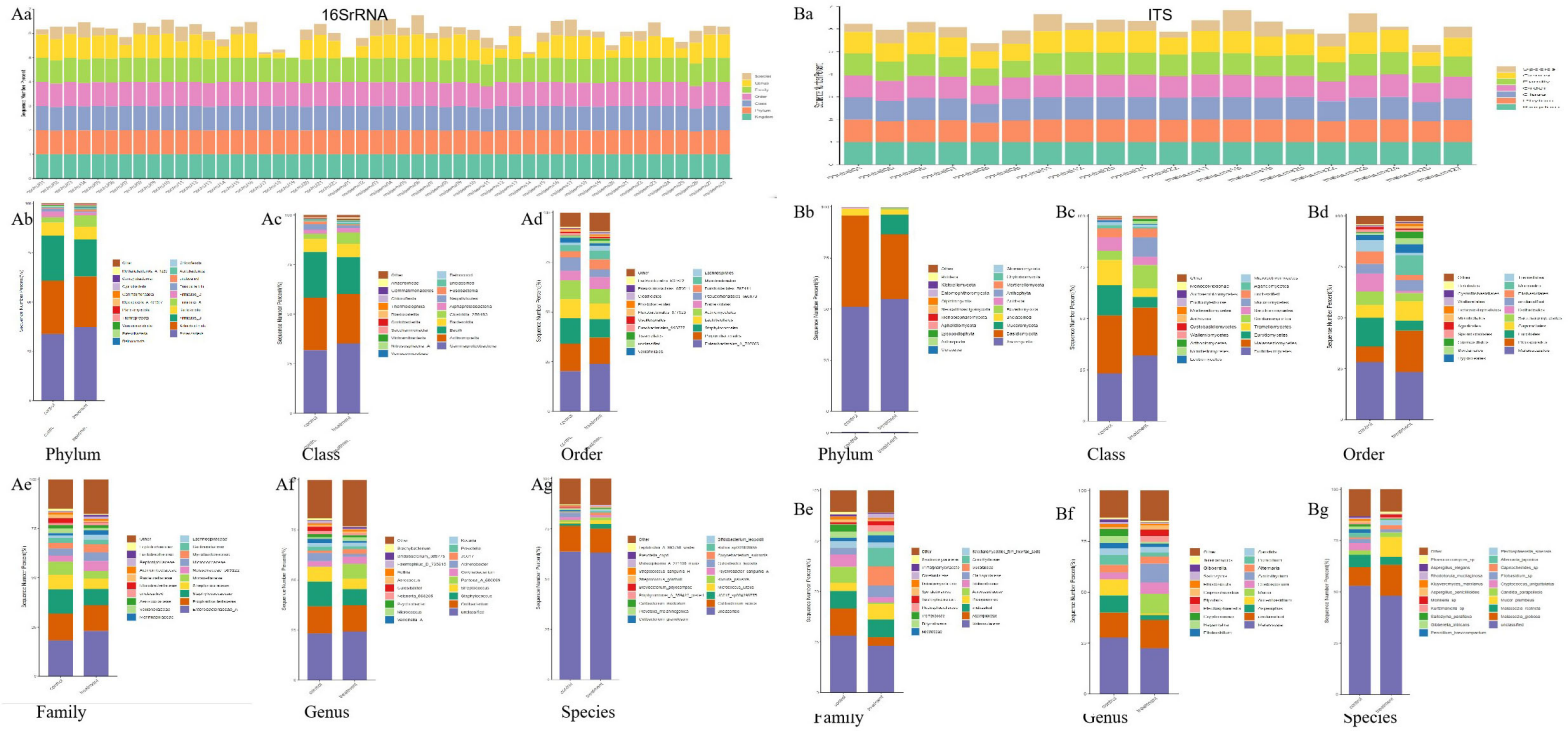
Bar charts of bacterial (16SrRNA) and fungal (ITS) sequence annotation degrees of each sample at each classification level. (**A**a) Bar charts of 16SrRNA sequence annotation degrees of each sample at each classification level in all; (**A**b–g) Bar plot of 16SrRNA sequence annotation at Phylum, Class, Order, Family, Genus, Species classification level; (**B**a) Bar charts of ITS sequence annotation degrees of each sample at each classification level; (**B**b–g) Bar plot of ITS sequence annotation at Phylum, Class, Order, Family, Genus, Species classification level.

Utilizing the Illumina Novaseq sequencing platform, bacterial (16SrRNA) and fungal (ITS) sequencing and data quality control optimization and analysis were performed on 50 volunteers (22 melasma patients and 28 healthy people without melasma). Bacterial species information was taxonomically analyzed according to the 97% similarity level, and a total of 17,704 valid sequences were obtained, and 2 Kingdom, 75 Phylum, and 165 Class,463 Order, 817 Family, 2,205 Genus, 2,545 Species, 17,703 OTUs were obtained by clustering operation (Figure 1). And 20 of the volunteers (11 melasma people, nine people without melasma) were classified as fungi, and 3,684 sequences were obtained, which were attributed to 10 Phyla, 25 Phylums, 50 Class, 140 Orders, 278 Families, 592 Genus, 896 Species, and 3,571 OTUs (Figure 1B). From the comparison, discrimination of genus diversity may had more difference.

#### 3.2.2 Community diversity analysis

##### 3.2.2.1 Alpha diversity analysis

By comparing and analyzing the Alpha diversity indices of skin flora in the melasma group and the control group (Tables 4, 5), it can be seen that the chao index of bacteria and fungi were on an increasing trend, and the abundance of skin flora in the melasma group was higher than that in the control group, but the difference was not statistically significant (*P* > 0.05). There was an increasing trend in shannon index and a decreasing trend in simpson index for bacteria and fungi, and the diversity of the melasma group was higher than that of the control group, but the difference was not statistically significant (*P* > 0.05), indicating similar community structures.

**TABLE 4:** Results of alpha diversity statistics (X ± S).

Microbiota	Alpha diversity index	Melasma	Control	*P*-value
Bacteria (*n* = 50)	Chao	656.89 ± 374.85	558 ± 406.81	0.097
	Shannon	4.59 ± 1.96	4.56 ± 1.97	0.907
	Simpson	0.76 ± 0.24	0.78 ± 0.25	0.769
Fungus (*n* = 20)	Chao	295.33 ± 179.25	274.45 ± 108.09	0.751
	Shannon	4.34 ± 2.1	5.03 ± 0.98	0.345
	Simpson	0.77 ± 0.28	0.91 ± 0.07	0.137

**TABLE 5:** PERMANOVA and ANOSIM results of group significance.

Class	PERMANOVA		ANOSIM	
	Pseudo-F	*P*-value	R	*P*-value
16SrRNA	1.153947	0.247	−0.022679	0.763
ITS	1.250199	0.083	0.116772	**0.037**

The bold value indicates that ANOSIM results of group have significant difference.

##### 3.2.2.2 Beta diversity analysis

**FIGURE 2:**
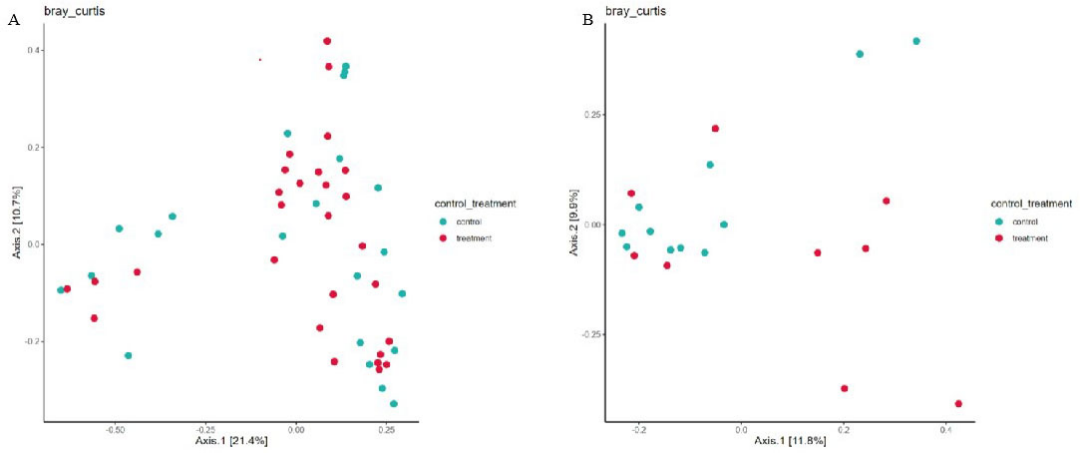
Principal coordinate anal (PCoA) plots of skin bacteria **(A)** and fungi **(B)** in the melasma group and the control group (genus level).

**FIGURE 3:**
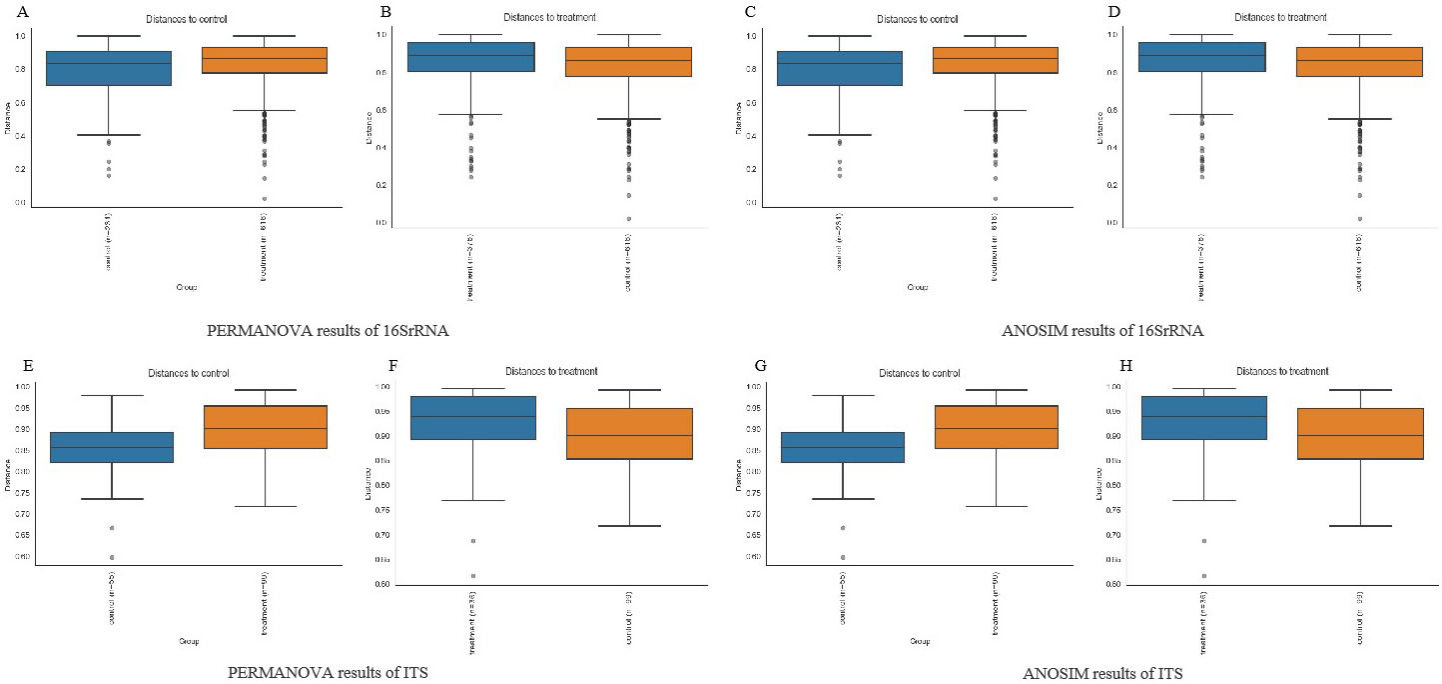
PERMANOVA and ANOSIM results of group significance pl*ots.* **(A,B)** Represent PERMANOVA results of bacterial (16SrRNA) group significance plots. **(C,D)** Represent ANOSIM results of 16SrRNA group significance plots. **(E,F)** Represent PERMANOVA results of fungal (ITS) group significance plots. **(G,H)** Represent ANOSIM results of ITS group significance plots.

In this study, PCoA analysis was used to investigate the similarity or difference in the composition of the sample communities (Figure 2). The results showed that the bacterial and fungal flora structure and composition of skin bacteria and fungi in melasma patients and the control population were basically the same, with a high degree of overlap, and could not be effectively distinguished (*p* > 0.05). Base on the result of PERMANOVA and ANOSIM of group significance plots, it was almost the same tendency while ANOSIM of ITS indicated the group difference between groups (Figure 3).

#### 3.2.3 Analysis of species differences between groups in genus level

As shown in Tables 5, 6, it can be seen that the facial skin bacteria in both samples had Cutibacterium, Staphylococcus, Streptococcus, and Acinetobacter as the dominant genera at the genus level. The comparison yielded that the proportion of dominant species such as Propionibacterium was basically the same, and the relative abundance of Staphylococcus, Streptococcus and Acinetobacter in the melasma group was greater than that in the control group, but there was no statistically significant difference (*P* > 0.05).

**TABLE 6:** Differences in relative abundance of skin flora between melasma and control groups at the genus level (X ± S).

Microbiota	Genus	Melasma (%)	Control (%)	*P*
Bacteria	Cutibacterium	13.99 ± 13.67	13.03 ± 18.83	0.494
	Staphylococcus	12.54 ± 15.82	8.08 ± 8.38	0.564
	Streptococcus	7.67 ± 9.92	5.12 ± 8.92	0.532
	Acinetobacter	2.94 ± 6.78	1.42 ± 3.03	0.784
	Corynebacterium	2.79 ± 2.78	3.61 ± 6.37	0.769
	Gulosibacter	2.22 ± 10.14	0.07 ± 0.29	0.921
	Veillonella_A	2.14 ± 3.09	0.98 ± 1.83	0.109
	Prevotella	2.12 ± 3.34	1.48 ± 3.46	0.564
	Neisseria_563205	1.71 ± 2.51	0.94 ± 2.67	0.233
	Psychrobacter	1.47 ± 5.00	1.04 ± 5.33	0.968
Fungus	Malassezia	27.69 ± 22.12	22.5 ± 22.02	0.552
	Aspergillus	8.36 ± 12.03	2.32 ± 2.99	**0.025**
	Aureobasidium	7.96 ± 12.93	0.90 ± 1.62	**0.012**
	Penicillium	4.96 ± 6.27	1.91 ± 2.93	0.331
	Alternaria	3.7 ± 7.18	3.56 ± 5.78	0.370
	Cryptococcus	3.5 ± 11.34	0.05 ± 0.1	0.175
	Naganishia	3.31 ± 4.64	0.93 ± 1.82	0.201
	Cladosporium	3.18 ± 2.42	5.63 ± 6.31	0.656
	Candida	3.15 ± 4.85	2.65 ± 3.58	0.882
	Filobasidium	2.76 ± 4.38	1.67 ± 2.16	0.603

The bold values indicate that the relative abundance of skin flora of Aspergillus and Aureobasidium between melasma and control groups at the genus level has significant difference.

On the other hand, skin fungi, were dominated by Malassezia, Aspergillus, Aureobasidium, and Penicillium. Aspergillus, and Aureobasidium were significantly higher in the melasma group than in the control group (*P* < 0.05). The dominant genera such as Malassezia spp. in the melasma group were also greater than the control group, but there was no statistical significance (*P* > 0.05).

### 3.3 Correlation analysis

**FIGURE 4:**
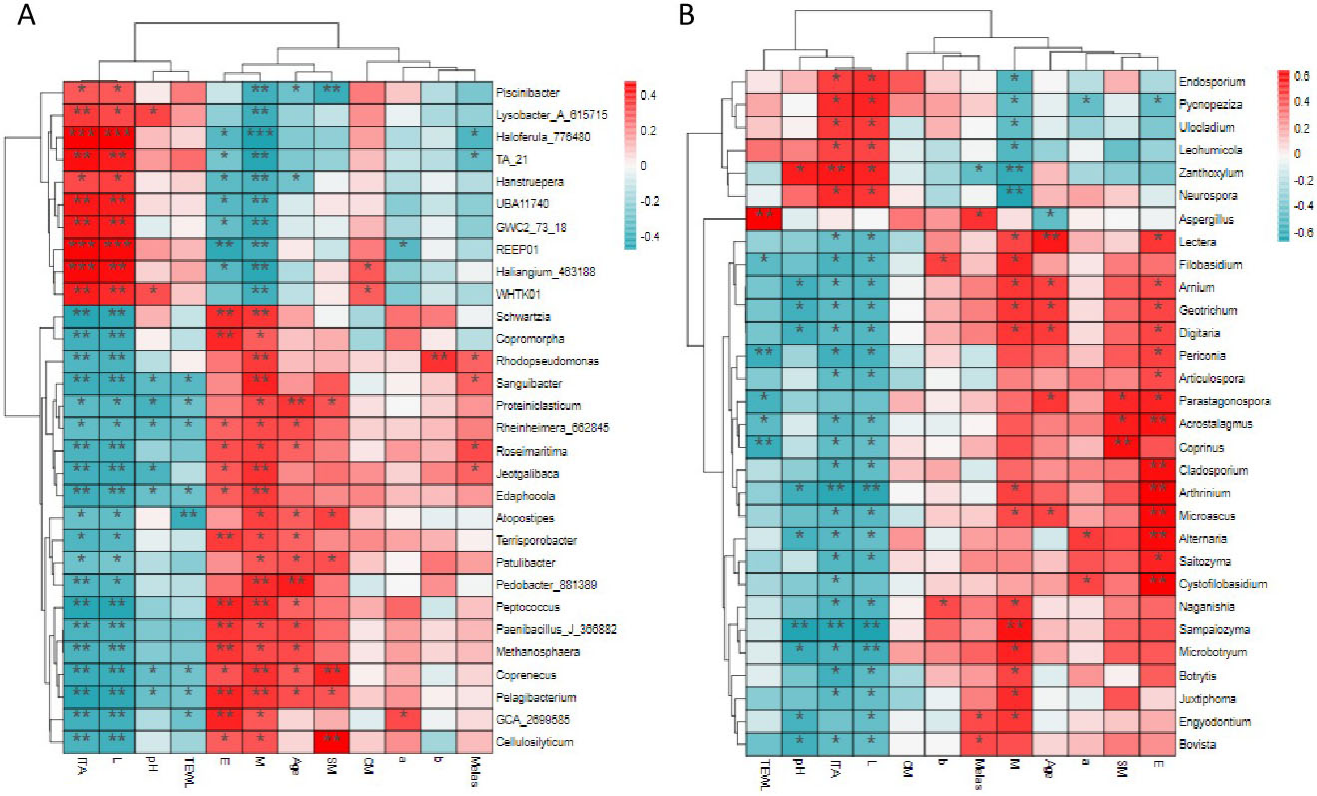
Correlation analysis of bacterial **(A)** and fungal **(B)** flora characteristics with skin parameters. *p < 0.05, **p < 0.01, ***p < 0.001.

The correlation analysis of bacterial and fungal flora characteristics and skin parameters showed that skin parameters such as ITA, TEWL, pH and L had a good correlation with REEP01 and other flora parameters (Figure 4). Haloferula_776480, REEP01, Haliangium_463188 in bacteria with ITA (*p* < 0.001), L parameters, TA_21, UBA11740, GWC2_73_18, WHTK01 and other parameters associated with ITA and L, Schwartzia, Copromorpha, and ITA and L (*p* < 0.01), Zanthoxylum with ITA and Lectera compared with Age, SM, and E in fungi (*p* < 0.01); Bacteria Rhodopseudomonas, Sanguibacter, Roseimaritima, Jeotgalibaca, Filobasidum, Naganishia, Aspergillus, and other fungi with the occurrence of Melas (*p* < 0.05) all had positive correlation, suggesting potential associations with melasma development (Figure 4).

Haloferula_776480 associated with M (*p* < 0.001), Piscinibacter, TA_21, UBA11740 associated with M, SM, Schwartzia, Copromorpha, E, M (*p* < 0.01); In fungi, Zanthoxylum, Neurospora, M, pH, etc., (*p* < 0.01); Haloferula_776480, TA_21 and other bacteria with Melas (*p* < 0.05), all had negative correlation (Figure 4).

### 3.4 ROC analysis

**FIGURE 5:**
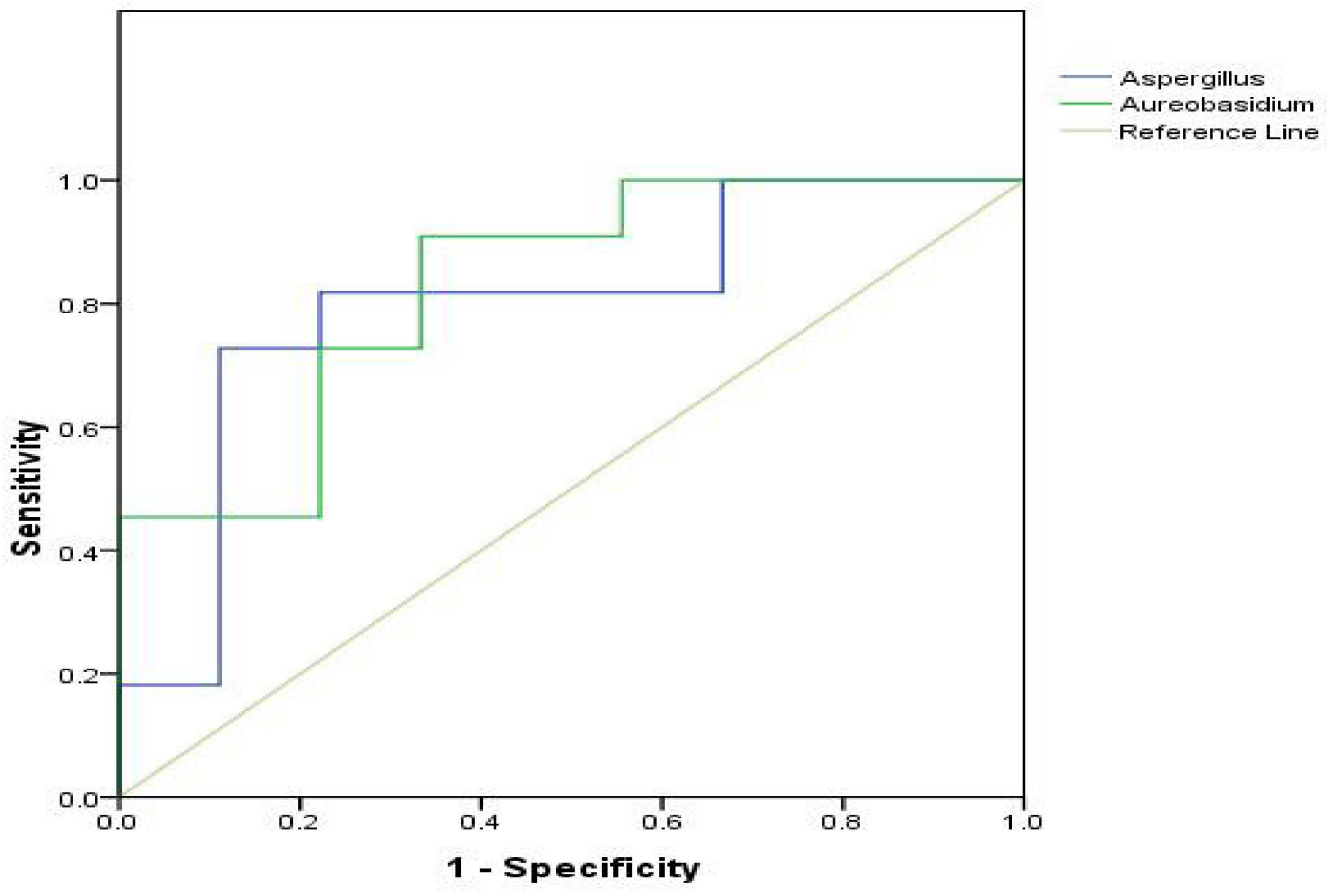
Receiver operating characteristic curve of melasma.

The ROC analysis assessing fungal such as Aspergillus, Aureobasidium as a predictor of melasma yielded an Area Under the Curve of 0.798, 0.828, indicating fair discriminatory ability (Figure 5, and Table 7).

**TABLE 7:** The result of receiver operating characteristic (ROC) curve for flora characteristics as a predictor of melasma.

Variables	Cutoff value	Sensitivity	Specificity	AUC (95% CI)
Aspergillus	0.0064	0.909	0.667	**0.798** (0.579–1.000)
Aureobasidium	0.0010	0.909	0.556	**0.828** (0.629–1.000)

The bold values indicate that receiver operating characteristic (ROC) curve for flora characteristics as a predictor of melasma indicates a moderate diagnostic value.

## 4 Discussion

This study characterized skin physiology and microflora in Tibetan melasma patients residing in high-altitude Lhasa. Key findings include a relatively high melasma prevalence (11.92%), distinct skin parameter alterations (lower pH, higher M), and specific fungal dysbiosis (Aspergillus, Aureobasidium enrichment), despite overall microbial community similarity to controls. Significant correlations and ROC curve link microflora features with skin parameters and melasma prevalence.

The average prevalence rate of melasma in the high altitude stood at 11.92%. This rate was lower than that in Shanghai, which is in the plain (13.16%) (Sun et al., 2022), as well as the results of similar pigmented skin disorders research in Lhasa (14.94% for melasma, 17.14% for high altitude facial telangiectasia, and 0.38% for vitiligo) (Xu et al., 2025). The underlying cause remains incompletely understood. Sample size limitations and stringent inclusion criteria may contribute to this variation. One probable reason is the small sample size of this study. Further research on population distribution and strict testing population condition screening is necessary to analyze the prevalence rate more precisely, which is limitations and future direction. Additionally, UV radiation is the primary factor that induces or exacerbates melasma (He et al., 2023). Different age groups demonstrated diverse trends in the incidence of melasma. This might be attributed to the high average altitude and intense ultraviolet radiation in Tibet, which lead to severe skin damage (Liu et al., 2021) and render the skin more susceptible to melasma, while how exact UVB radiation dose exert its effect is uncertain. The prevalence rate in females was significantly higher than that in males, and it peaked in the 31–40 age group, which was also in line with previous research on the incidence of melasma in plain areas (Sun et al., 2022). The pH value of melasma patients in high altitude areas was notably lower than that of control group, while the M value was significantly higher. This indicates that the skin of melasma patients is more acidic and has greater melanin accumulation. Compared with the E value, a value, and b value of skin parameters reported in the literature, no differences were observed (Sun et al., 2022). This could be due to differences in the study population or the discrepancy between the baseline data of skin parameters in high altitude environments and those in the plain, indicating that skin parameter can only partly predict the occurrence and development of melasma and may reflect unique high-altitude skin adaptations or baseline differences.

Based on the characteristics of the flora, the latest research (Gao et al., 2024) reveals that the imbalance of the skin microflora is linked to the onset or worsening of melasma. This is mainly because microorganisms can influence the skin’s homeostasis and inflammatory processes. Integrating the results of microbial sequencing analysis, the richness, diversity, flora structure, and composition of skin bacteria and fungi communities in this study were basically consistent with those of people without melasma, which was relatively consistent with previous studies. The inter group analysis of different genus showed that Propionibacterium, Staphylococcus, and Streptococcus were the dominant bacterial genera, while Malassezia, Aspergillus, Chrysobasidiomyces, and Penicillium were the dominant fungal genera. However, some research has shown that the abundances of Collinsella, Actinomyces, Parabacteroides, Bacteroides, Paraprevotella, Blautia, and Roseburia in the melasma group were significantly different from those in the healthy group (Mekadim et al., 2022). Moreover, previous studies have indicated that Lactobacillus, Clostridium, and Corynebacterium are the most distinct genera in the microbiome of healthy skin, while Clostridium, Eupereira, Staphylococcus, Streptococcus, and Bacteroides are abundant in the microbiome of melanoma tissues, respectively (Mekadim et al., 2022). In terms of fungal genera, there were differences in flora composition. Specifically, in the melasma group, the abundances of Aspergillus and Aureobasidium were greater enriched than those in the control group (*P* < 0.05). This came to the known result that Kojic add was a fungal metabolite, mainly produced by Aspergillus fungi and was commercially available as an effective whitening agent in the treatment of hyperpigmentary conditions, like melasma (Da Silva et al., 2013; Rodrigues et al., 2014; Shenoi et al., 2005) and Tinea nigra (TN) was a superficial fungal infection caused by the melanized fungus Aureobasidium melanogenum and so on, characterized by irregular dark patches, typically on the palms (Amaral et al., 2024; Sanchez-Romero et al., 2025; Watanabe and Hashimoto, 2023). Namely, Aspergillus species produce kojic acid, a recognized depigmenting agent used in melasma treatment, while Aureobasidium melanogenum causes tinea nigra, characterized by dark macules. Yet, it lack some deeply experimental verification and product guarantee. This was consistent with the flora reported in existing literature, yet insufficient to suggest that the occurrence of melasma was inconsistent with the disturbance of the microbial flora. This might be due to the high altitude environmental stress and the rich biodiversity. However, there may be other factors such as hygiene habits, cosmetic use, UV exposure influencing the result. This implies that melasma exhibits prominent fungal flora characteristics, which could potentially represent potential microbial targets for intervention and serve as a biological target or a key strain for control (Grimes et al., 2022), which will benefit for future research direction and patient’s burden from clinical perspectives, and is of vital importance regulation of diseases from regulatory perspectives.

Regarding the correlation analysis, the characteristics of the flora were significantly correlated with ITA, TEWL, pH, E, M, and SM, all of which may further support a link between the microbiome and melasma pathogenesis. Notably, skin parameters such as pH and M, which were closely related to the formation of melasma in the high altitude, were strongly correlated with the flora. Some studies have employed microbial regulatory agents and pH balancing agents, such as local probiotics and prebiotics, which have effectively achieved the dilution effect on melasma, indirectly demonstrating the correlation between microbial colony characteristics and pH. Currently, only the relationship between gut microbial dysbiosis and vitiligo has been preliminarily explored (Grimes et al., 2022). Additionally, the integrity of the skin barrier and local pH regulation, which are crucial for maintaining a balanced microbiome, may also contribute to hyperpigmentation. What’s more, Aspergillus with the occurrence of Melas (*p* < 0.05) all had positive correlation and the ROC analysis assessing Aspergillus, Aureobasidium as a predictor of melasma possessed a satisfied AUC, which could benefit for indicating the occurrence of the disease. Despite the plethora of treatment modalities for melasma, there is a high risk of recurrence and often incomplete treatment, justifying the primary care physician’s role in early diagnosis and prevention (Hang and Lim, 2025; Pandya et al., 2025; Poudyal et al., 2025). Various modalities include corticosteroid triple combinations, hydroquinone (HQ), and tretinoin, superficial chemical creams, nonsteroidal demelanizing creams, (trichloroacetic acid, GA, lactic acid, and kojic acid, AA, salicylic acid, etc..), lasers (Alexandrite laser, ruby laser, Q-switched Nd: YAG laser, Fraxel laser, and Er: YAG laser), and intense pulsed light (IPL), but their thearpy results are poor. This give us new insight perspective through microbial therapy or combined therapy, which will step to the forefront of the world (Chen et al., 2024; Dairov et al., 2025; Sharma et al., 2024). And also, more than 1% of the general population suffers from melasma, which is defined as a failure of melanogenesis resulting in persistent hypermelanosis of the skin. Patients with melasma revealed to have increased oxidative stress marker levels because melasma activates inducible nitric oxide, which in turn induces reactive oxygen species. Although several therapy options, including tyrosinase inhibitors, anti-inflammatory steroids, and topical retinoids, have been employed more often in studies, darker-complexioned individuals have not shown a noticeable improvement in melasma pigmentation (Aung et al., 2024; de Freitas et al., 2023; Liang et al., 2023). Given from clinical applications and real-world translation, this research maybe give a new prosperity towards curing patients not only melasma, but also for hyperpigmentation. Targeting the microbiome offers a novel therapeutic avenue, especially relevant given the role of oxidative stress in melasma and the limited efficacy of conventional treatments in darker skin.

In conclusion, the study uncovers that the skin of Tibetan melasma patients residing in Lhasa, Tibet manifests remarkable pH sensitivity, augmented melanin content, and a diverse bacterial community structure despite overall microbiome structural similarity to that of the control population. A robust correlation exists between the diversity of the bacterial community structure and skin parameters and ROC analysis curve acting as a predictor of melasma. The predictive potential of specific fungi offers a novel approach for early detection. Thereby it was furnished a novel theoretical foundation for more in-depth skin management and the application of prediction, prevention of melasma. Nevertheless, the sample size of this research remains relatively small, and there are still deficiencies in the data mining of the functional metabolic pathways of bacteria and fungi, as well as in the exploration of prevention, prediction and control mechanisms. Future investigations ought to concentrate on expanding the sample space and establishing quantitative mechanistic correlation relationships and mechanisms, and exploring targeted microbiome modulation for skin health restoration in high-altitude environments. To build upon the novel associations uncovered between skin physiological parameters, fungal microbiome composition, and melasma prevalence in high-altitude Tibetan populations, future research would focus on conducting longitudinal or interventional studies that directly test the effect of targeted microbiome modulation on melasma outcomes. For example, randomized controlled trials utilizing topical probiotics, prebiotics, or antifungal agents that specifically alter skin microbial profiles—particularly those genera identified as predictive (e.g., Aspergillus, Aureobasidium)—could determine whether clinical improvement or prevention of melasma is achievable by modifying the skin microenvironment. Such studies would provide causal evidence and potentially open new avenues for personalized treatment approaches in dermatology, especially in environmentally unique populations. This approach also has the potential to offer valuable insights for the development of targeted and personalized therapies aimed at restoring and maintaining the skin health of individuals in high-altitude regions.

## Data Availability

The data presented in the study are deposited in the NCBI repository, accession numbers PRJNA1390492 and PRJNA1391686.

## Footnotes

1
https://docs.qiime2.org/2019.1/

2
https://www.bioincloud.tech/
